# Maintenance of the virulence plasmid in *Shigella flexneri* is influenced by Lon and two functional partitioning systems

**DOI:** 10.1111/mmi.14225

**Published:** 2019-03-22

**Authors:** Gareth McVicker, Sarah Hollingshead, Giulia Pilla, Christoph M. Tang

**Affiliations:** ^1^ Sir William Dunn School of Pathology University of Oxford South Parks Road Oxford OX1 3RE UK; ^2^ Present address: School of Science and Technology Nottingham Trent University Nottingham UK

## Abstract

Members of the genus *Shigella *carry a large plasmid, pINV, which is essential for virulence. In *Shigella flexneri*, pINV harbours three toxin‐antitoxin (TA) systems, CcdAB, GmvAT and VapBC that promote vertical transmission of the plasmid. Type II TA systems, such as those on pINV, consist of a toxic protein and protein antitoxin. Selective degradation of the antitoxin by proteases leads to the unopposed action of the toxin once genes encoding a TA system have been lost, such as following failure to inherit a plasmid harbouring a TA system. Here, we investigate the role of proteases in the function of the pINV TA systems and demonstrate that Lon, but not ClpP, is required for their activity during plasmid stability. This provides the first evidence that acetyltransferase family TA systems, such as GmvAT, can be regulated by Lon. Interestingly, *S. flexneri* pINV also harbours two putative partitioning systems, ParAB and StbAB. We show that both systems are functional for plasmid maintenance although their activity is masked by other systems on pINV. Using a model vector based on the pINV replicon, we observe temperature‐dependent differences between the two partitioning systems that contribute to our understanding of the maintenance of virulence in *Shigella* species.

## Introduction

Plasmids play a critical role in enabling bacteria to adapt to specific environments and stresses. Genes encoded on plasmids can confer resistance to antibiotics and other toxic compounds, while virulence plasmids in bacterial pathogens mediate invasion and survival within specific niches during the development of disease. For example, the four species of *Shigella* have emerged from commensal *Escherichia coli* following the acquisition of a large ~210 kb plasmid, pINV (Pupo *et al.*, 2000). pINV is critical for *Shigella *virulence as it carries a 30 kb pathogenicity island (PAI) that encodes a Type III Secretion System (T3SS), a molecular syringe that delivers effector proteins directly from the bacterium into human cells, mediating cell invasion (Sansonetti, 1991; Buchrieser *et al.*, 2000). In addition, pINV carries other virulence genes involved in adhesion and actin‐mediated motility, such as *icsA* (Bernardini *et al.*, 1989; Brotcke Zumsteg *et al.*, 2014), as well as T3SS effector proteins encoded outside the PAI, including *Shigella* enterotoxin 2 (Farfan *et al.*, 2011) and the multicopy *ipaH* genes (reviewed by Ashida and Sasakawa, 2015).

Large virulence plasmids can impose a significant burden on the replication and fitness of their bacterial host, as in the case of *Shigella *(Sasakawa *et al.*, 1986). pINV is likely present in a single copy as suggested by its replicon (Buchrieser *et al.*, 2000) and next‐generation sequencing read depth relative to the chromosome (unpublished data). During growth in the laboratory, plasmid instability can result in complete loss of pINV, internal plasmid deletions and chromosomal integration of pINV, leading to absent or reduced expression of the T3SS (Sasakawa *et al.*, 1986; McVicker and Tang, 2016; Pilla *et al.*, 2017). The plasmid has several mechanisms to ensure its retention in a growing bacterial population. These mechanisms include toxin‐antitoxin (TA) modules that mediate post‐segregational killing (PSK; Sayeed *et al.*, 2005; Winther and Gerdes, 2011; McVicker and Tang, 2016), as well as uncharacterised partitioning systems belonging to the ParAB and ParMR families (Buchrieser *et al.*, 2000).

TA systems employ a stable, toxic protein that interferes with a key aspect of bacterial viability, such as translation or DNA replication (Bernard and Couturier, 1992; Winther and Gerdes, 2011), resulting in growth arrest and/or death. Protein antitoxins of type II TA systems counteract the effect of their cognate toxins, usually through physical sequestration (reviewed by Harms *et al.*, 2018). However, antitoxins are labile as they are targeted for degradation by cellular proteases such as Lon and ClpP (van Melderen *et al.*, 1996; Winther and Gerdes, 2012), so need to be continually replenished by *de novo* expression from the TA operon. If the genes encoding the TA system are lost, for example, due to failure to segregate the plasmid or following a recombination event (Wozniak *et al.*, 2009; Pilla *et al.*, 2017), antitoxin levels fall and the unopposed activity of the toxin prevents the growth of plasmid‐free daughter cells, leading to PSK. As a consequence, the activity of type II TA systems, which are abundant on virulence plasmids, is critically dependent on the recognition and cleavage of the antitoxin by cellular proteases. The spontaneous appearance of strains lacking a plasmid reflects a defect in or absence of PSK.

Plasmid partitioning systems are closely related to chromosomal partitioning systems and function by promoting the physical separation of newly replicated plasmids into the two daughter cells during replication. Partitioning occurs either by separating plasmid DNA bound by partitioning proteins along a localised concentration gradient (type I, ParAB systems), or by the assembly of dynamic, actin‐like filaments that push each plasmid away from the mid‐cell (type II, ParMR systems) (mechanisms reviewed by Ebersbach and Gerdes, 2005). Interestingly, VirB, which regulates genes both on the *Shigella flexneri* PAI (Watanabe *et al.*, 1990) and elsewhere on the virulence plasmid (Wing *et al.*, 2004; Le Gall *et al.*, 2005; Weatherspoon‐Griffin *et al.*, 2018), has significant homology to proteins involved in partitioning (i.e*.* ParB and SopB encoded by plasmids P1 and F respectively) (Watanabe *et al.*, 1990). Therefore, partitioning systems on virulence plasmids may have functions aside from their canonical role in plasmid segregation. *S. flexneri* pINV is predicted to encode two partitioning systems, ParAB and StbAB (Buchrieser *et al.*, 2000), which are type I and type II partitioning systems respectively. *Shigella* ParAB shares 65.7% sequence identity with the well‐characterised type I partitioning system on the *E. coli* P1 plasmid (Lobocka *et al.*, 2004). StbAB is more distantly related to the ParMR system of the R1 plasmid (32.8% average amino acid identity to ParMR), yet retains a conserved aspartic acid at position 173 that is necessary for the ATPase activity and function of ParM (Jensen and Gerdes, 1997). To date, the activity of these partitioning systems on pINV has not been investigated, although the function of *S. flexneri* ParAB has been confirmed in *E. coli *using a mini‐P1 vector (Sergueev *et al.*, 2005).

Of the four *Shigella* species that cause bacillary dysentery, *S. flexneri* is the most prevalent, accounting for around 80% of all cases worldwide (Kotloff *et al.*, 1999). *S. flexneri* pINV encodes three functional TA systems, MvpAT, CcdAB and GmvAT. MvpAT is a member of the VapBC family and contributes to plasmid maintenance at temperatures found in the environment and the human intestine (Sayeed *et al.*, 2005; McVicker and Tang, 2016). Given the extensive studies in other bacteria on this and related TA systems (reviewed by Arcus *et al.*, 2011), here we refer to MvpAT as VapBC, in which VapB is the antitoxin, and VapC is a toxic ribonuclease. CcdAB employs a DNA gyrase inhibitor, CcdB, as the toxin with CcdA acting as its antidote (Bahassi *et al.*, 1999); this TA system does not appear to play a major role in plasmid stability in *S. flexneri *(McVicker and Tang, 2016). The third TA system, GmvAT, utilises an acetyltransferase toxin that prevents translation, and contributes significantly to pINV stability at 21°C (McVicker and Tang, 2016). Acetyltransferase TA systems are becoming more widely characterised (Jurenas *et al.*, 2017; Qian *et al.*, 2018; Rycroft *et al.*, 2018; Wilcox *et al.*, 2018). Interactions between TA and partitioning systems are poorly defined and the subject of speculation. For example, it has been proposed that *S. flexneri* VapBC and P1 ParAB act together to optimise plasmid stability whilst alleviating the growth deficit incurred by PSK (Brendler *et al.*, 2004).

Here, we undertook a systematic analysis of TA and partitioning systems on *S. flexneri* pINV. We demonstrate that the TA systems responsible for pINV stability in *S. flexneri* are governed by the activity of Lon protease, while ClpP has no role in the function of these systems. To elucidate the contribution of individual PSK and partitioning systems to plasmid stability, we constructed a model vector harbouring the replicon from pINV so we could study the role of each TA module and partitioning system in isolation. Our results demonstrate that the partitioning systems ParAB and StbAB do not have a major impact on pINV stability in *S. flexneri*, irrespective of the presence of the TA systems, even though our model vector shows that both ParAB and StbAB are functional and influenced by the ambient temperature. Our findings further current understanding of the maintenance of virulence in important pathogens such as *Shigella*.

## Results

### pINV TA systems are dependent on the Lon protease for plasmid stability

**Figure 1:**
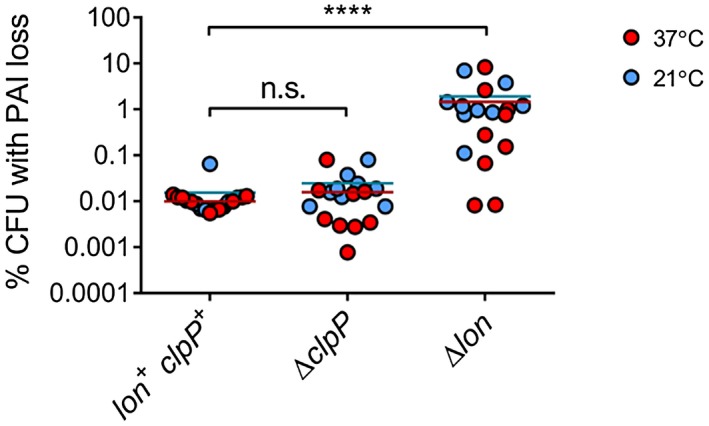
The effect of deleting *clpP* or *lon* on PAI loss in *S. flexneri. *PAI loss measured by *sacB‐neo^R^* assay in strains with protease gene deletions as indicated, and grown at 37 and 21°C for approximately 25 generations. ^****^ *p* < 0.0001; n.s. not significant by two‐way ANOVA with Tukey multiple comparisons test (*n* = 9 colonies from three independent experiments). [Colour figure can be viewed at wileyonlinelibrary.com].

Cellular proteases, such as Lon and ClpP, specifically degrade antitoxins from type II TA systems to effect PSK or bacterial entry into a persistent state (reviewed by Muthuramalingam *et al.*, 2016). Therefore, we investigated the contribution of the Lon and ClpP proteases to pINV stability in *S. flexneri* using a dual marker system as previously described (McVicker and Tang, 2016). Introduction of a *sacB‐neo^R^* cassette into *mxiH* enables selection and enumeration of bacteria which have lost the T3SS PAI (through their ability to grow on media containing sucrose) and bacteria that retain this region (by their resistance against kanamycin); this allows quantification of the loss of the T3SS PAI with an error rate of <1% (McVicker and Tang, 2016). We deleted the chromosomal copies of *lon* or *clpP* in *S. flexneri *harbouring *mxiH*:*sacB*::*neo* ^R^, and examined the stability of pINV during approximately 25 generations of growth. We found that pINV stability decreased by over two orders of magnitude at 37°C and at 21°C in a *lon* mutant compared to the parental strain (Fig. 1, *p* < 0.0001). In contrast, deletion of the gene encoding ClpP, which has been implicated in TA function previously (Lehnherr and Yarmolinsky, 1995; Aizenman *et al.*, 1996; Donegan *et al.*, 2010), did not significantly alter the stability of pINV (Fig. 1). We measured plasmid loss over a fixed number of generations (cell doublings) to take into account any differential growth rate of the mutants.

**Figure 2:**
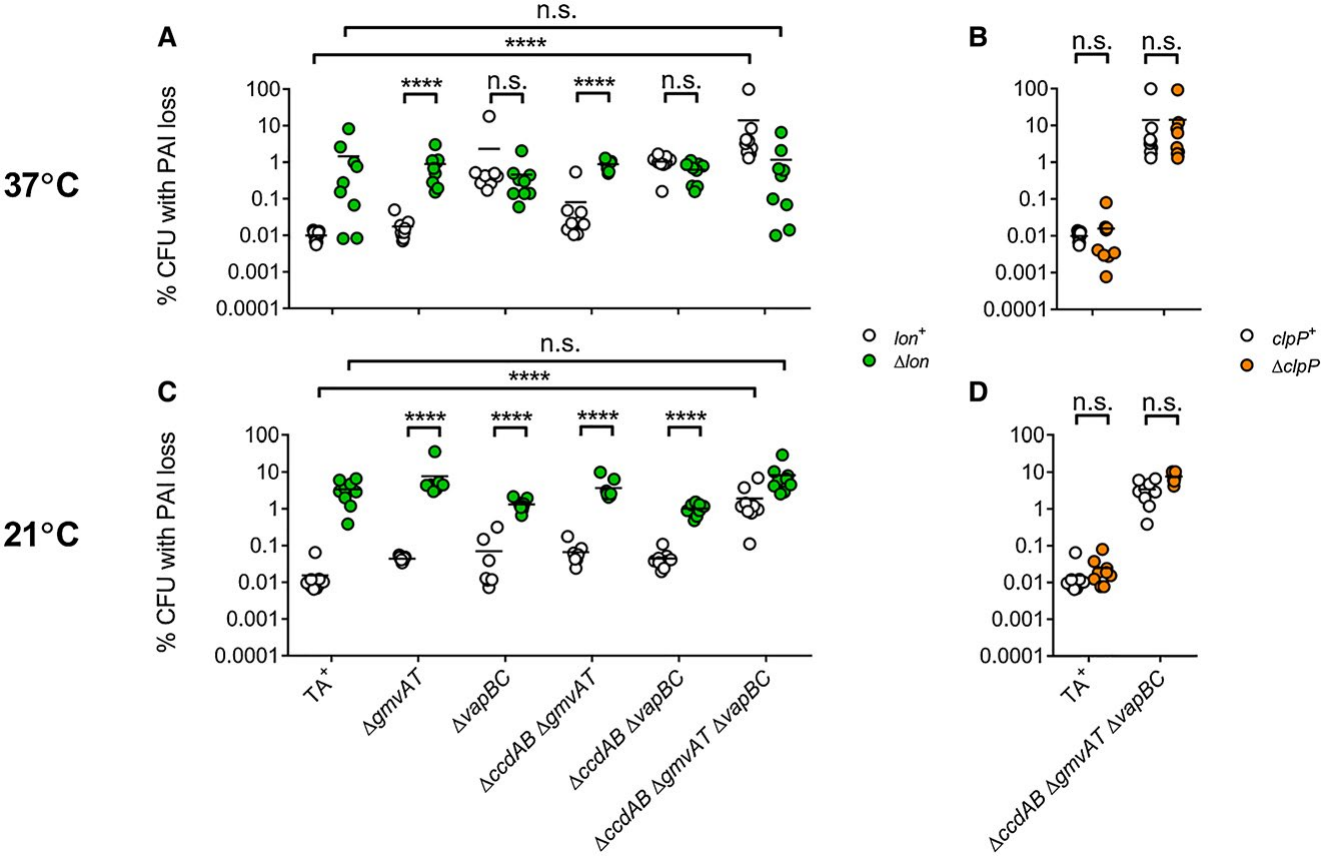
The effect of Lon and ClpP on individual TA systems present on *S. flexneri* pINV. Assays were performed (A) in the presence/absence of *lon *at 37°C, (B) in the presence/absence of *clpP *at 37°C, (C) in the presence/absence of *lon *at 21°C, or (D) in the presence/absence of *clpP *at 21°C. PAI loss measured by *sacB‐neo^R^* assay in strains grown at 37°C and 21°C for approximately 25 generations. Relevant statistical comparisons are indicated. ^****^ *p* < 0.0001; n.s. not significant by two‐way ANOVA with Tukey multiple comparisons test (*n* = 9 colonies from three independent experiments). [Colour figure can be viewed at wileyonlinelibrary.com].

Studies of the fate of *Salmonella enterica *VapB in *E. coli* indicate that this antitoxin is degraded by the Lon protease (Winther and Gerdes, 2012). Therefore, deletion of *lon* in *S. flexneri *should abrogate the contribution of VapBC to pINV stability. Consistent with this, in every pINV derivative containing VapBC at 37°C, plasmid stability was significantly reduced when *lon* was deleted (Fig. 2A, *p* < 0.0001). Furthermore, in the absence of VapBC, deletion of *lon *had no effect on pINV stability at 37°C (Fig. 2A, *p* = 0.5788), indicating that the effect of Lon at this temperature is due to its influence on VapBC.

VapBC is dispensable for pINV stability at 21°C (McVicker and Tang, 2016). However, deletion of *lon* also significantly reduced plasmid stability at this temperature, irrespective of the presence of VapBC (Fig. 2C, *p* < 0.0001), indicating that Lon acts through a different TA system at this temperature. Since GmvAT is sufficient to stabilise pINV at temperatures found outside the human host (McVicker and Tang, 2016), we next tested the impact of Lon on this TA system. We found that the stability of pINV containing *gmvAT* as the sole active TA system was reduced by the deletion of *lon* at 21°C (Fig. 2C, *p* < 0.0001), the temperature at which GmvAT functions, but not at 37°C (Fig. 2A, *p* = 0.6545). Crucially, plasmid stability in a *lon*‐negative background was not significantly affected by the removal of all three TA systems (VapBC, GmvAT and CcdAB) at either temperature (Fig. 2A and C, *p* > 0.092), in contrast to the *lon*‐positive background in which deletion of the three TA systems results in a marked decrease in pINV stability at both temperatures (Fig. 2A and C, *p* < 0.0001). Deletion of *clpP* did not affect pINV stability at 37 or 21°C (Fig. 2B and D, *p > *0.23). Taken together, these results demonstrate that Lon affects the activity of both VapBC and GmvAT in *S. flexneri*; this is the first example of Lon affecting a TA system, such as GmvAT, containing a toxic acetyltransferase.

### The ParAB partitioning system contributes to pINV maintenance

**Figure 3:**
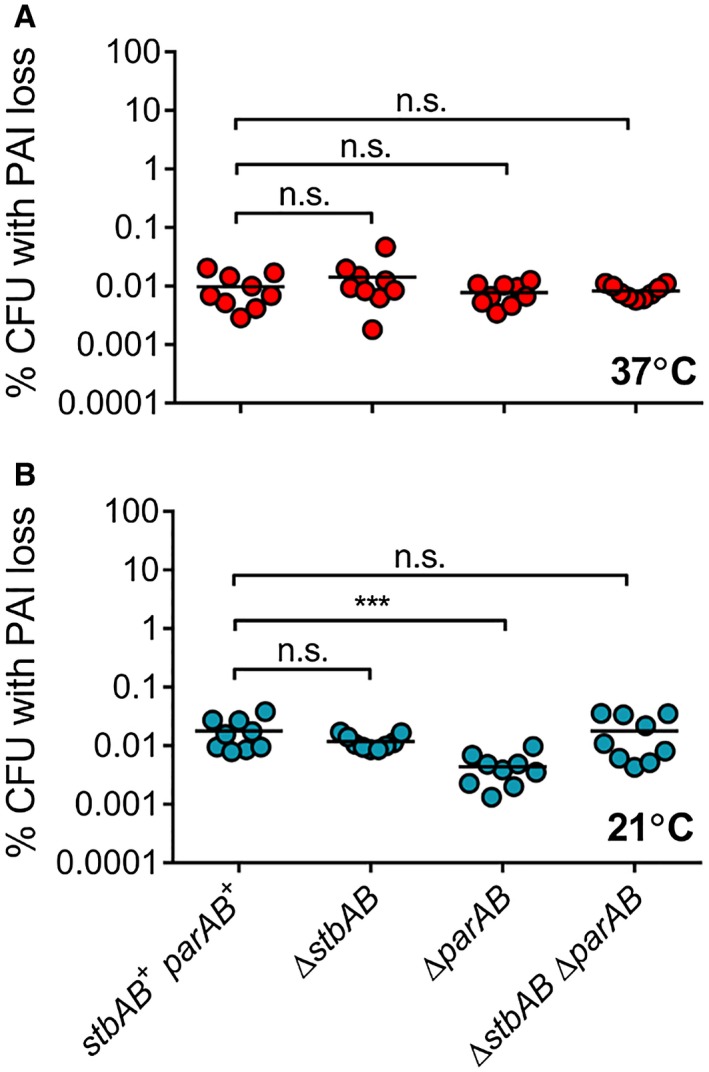
The combined effect of partitioning systems ParAB and StbAB on the stability of *S. flexneri* pINV. PAI loss measured using *sacB‐neo^R^* in strains carrying the partitioning systems or deletions as indicated (Δ*parAB*/Δ*stbAB*), grown at 37°C (A) and 21°C (B) for approximately 25 generations. ^***^ *p* < 0.001; n.s. not significant by two‐way ANOVA with Tukey multiple comparisons test (*n* = 9 colonies from three independent experiments). [Colour figure can be viewed at wileyonlinelibrary.com].

**Figure 4:**
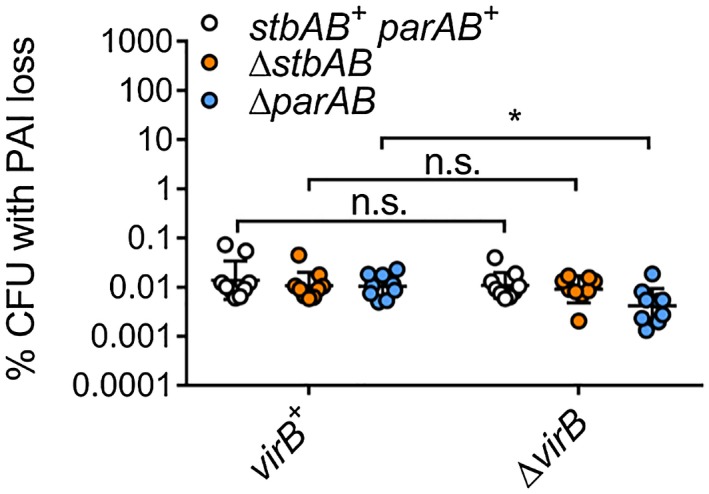
The effect of VirB on *S. flexneri* pINV stability in the presence and absence of ParAB and StbAB. PAI loss was measured using *sacB‐neo^R^* in strains with and without StbAB (*stbAB^+^/*Δ*stbAB*) or ParAB (*parAB^+^/*Δ*parAB*), in the presence or absence of VirB (*virB* ^+^/ΔvirB respectively) following growth at 37°C (A) or 21°C (B) for approximately 25 generations. ^*^ *p* < 0.05; n.s. not significant by two‐way ANOVA with Sidak multiple comparisons test (*n* = 9 colonies from three independent experiments). [Colour figure can be viewed at wileyonlinelibrary.com].

*Shigella flexneri *pINV contains two potential partitioning systems, ParAB and StbAB. *stbAB *is predicted to encode an uncharacterised ParMR‐related partitioning system (42.5% identity to ParM and 23.1% identity to ParR of the *E. coli* R1 plasmid, Supplementary Fig. 1; Gerdes and Molin, 1986). Nothing is known about the contribution of StbAB to pINV stability. Therefore, we assessed the impact of StbAB on plasmid stability by deleting *stbAB* from *S. flexneri *pINV. There was no difference in the stability of the plasmid with or without StbAB at 37 or 21°C, measured using the *sacB‐neo^R^*assay (Fig. 3, *p* > 0.63). As there could be functional redundancy between StbAB and ParAB, we constructed a double *parAB*/*stbAB* mutant and examined its effect on plasmid stability at 37 and 21°C relative to the single mutants (Fig. 3). Removal of *parAB* or both partitioning systems did not significantly destabilise the plasmid irrespective of temperature (*p* > 0.74). However, at 21°C, pINV was significantly more stable (*p* = 0.0001) in the *parAB* mutant compared with the wild‐type strain, and removal of *stbAB* in the *parAB* mutant restored wild‐type levels of stability (Fig. 3B). Additionally, the T3SS regulator VirB has homology with the partitioning protein, ParB (Watanabe *et al.*, 1990). Therefore, we analysed the effect of deleting *virB* in the presence and absence of the other putative partitioning systems at 37°C; the absence of VirB does not significantly decrease pINV stability under these conditions (Fig. 4, *p* < 0.85). Deletion of *parAB* does, however, increase stability in the strain lacking VirB (Fig. 4, *p* = 0.0228), consistent with its effect at 21°C (Fig. 3, discussed above), given that *virB* is not expressed at this temperature (Tobe *et al.*, 1995).

**Figure 5:**
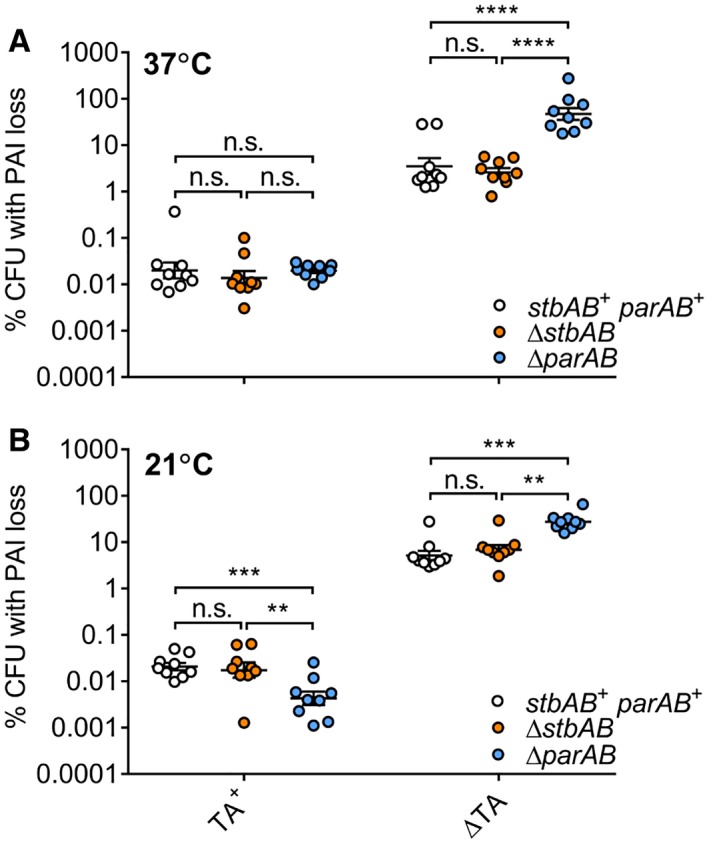
The effect of the putative partitioning systems on *S. flexneri* pINV stability in the presence and absence of MvpAT, GmvAT and CcdAB. PAI loss was measured using *sacB‐neo^R^* in strains with and without StbAB (*stbAB^+^/*Δ*stbAB*) or ParAB (*parAB^+^/*Δ*parAB*), in the presence or absence of the three TA systems (TA^+^/ΔTA respectively) following growth at 37°C (A) or 21°C (B) for approximately 25 generations. ^****^ *p* < 0.0001; ^***^ *p* < 0.001 ^**^ *p* < 0.01; n.s. not significant by two‐way ANOVA with Tukey multiple comparisons test (*n* = 9 colonies from three independent experiments). [Colour figure can be viewed at wileyonlinelibrary.com].

It is possible that the effect of partitioning systems on plasmid maintenance is masked by the presence of the TA systems on pINV, which could eliminate bacteria following defective plasmid segregation through PSK. Therefore, we also deleted *stbAB *and *parAB* from a plasmid lacking the TA systems, VapBC, CcdAB and GmvAT. Again, *stbAB* had no effect on plasmid stability even in the absence of these TA systems (Fig. 5, *p* > 0.74). In contrast, while deletion of *parAB* did not reduce pINV stability in the presence of the TA systems, the stabilising effect of this partitioning system at 37 and 21°C became evident in the absence of the TA systems (Fig. 5, *p* ≤ 0.0001).

Taken together, these results indicate that ParAB is a functional partitioning system that operates at 37 and 21°C and that StbAB contributes to plasmid stability at 21°C in the absence of ParAB. However, we could not examine the contribution of *stbAB* in the absence of *parAB* and the three TA systems together, as pINV became increasingly unstable with each deletion, and we were not able to generate the necessary strain despite multiple attempts.

### Construction of a model vector to assess factors contributing to plasmid stability

**Figure 6:**
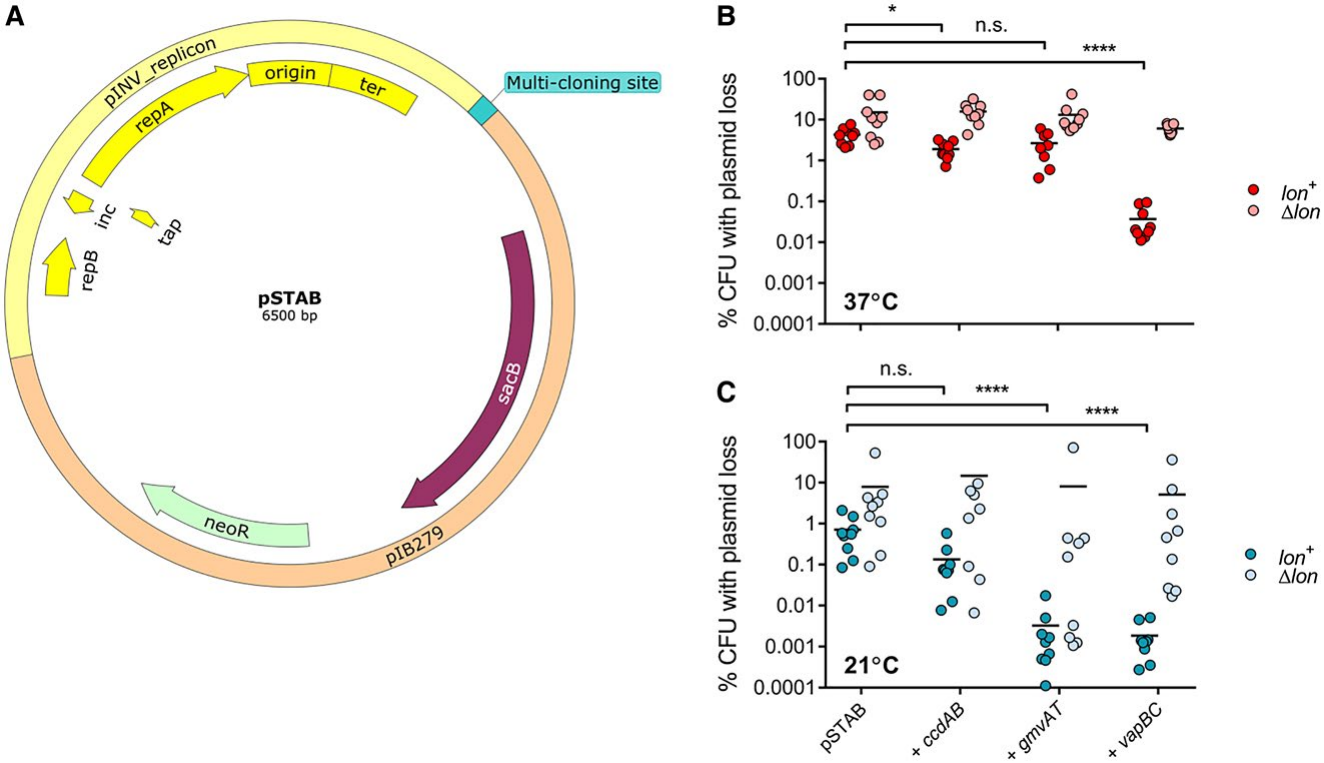
The effect of TA systems on pSTAB stability in *S. flexneri‐lacking *pINV. Loss of pSTAB (map in panel A) measured by *sacB‐neo^R^* assay with variants carrying either no insertion, VapBC, CcdAB or GmvAT, and grown at 37°C (B) or 21°C (C) in the presence (dark circles) or absence (light circles) of *lon* for approximately 25 generations. ^****^ *p* < 0.0001; ^*^ *p* < 0.05; n.s. not significant by one‐way ANOVA with Dunnett multiple comparisons test (*n* ≥ 8 colonies from at least three independent experiments). [Colour figure can be viewed at wileyonlinelibrary.com].

The impact of partitioning and PSK mechanisms on a large element such as pINV can be difficult to define given the multiplicity of maintenance systems, the likelihood of redundancies and interdependencies, and the presence of insertion sequences which allow for chromosomal integration (Pilla *et al.*, 2017). Therefore, we generated a vector, pSTAB, to enable analysis of the effect of individual systems in isolation. The replicon of *Shigella *pINV is a RepFIIA‐like element that is sufficient for plasmid propagation and incompatibility (Silva *et al.*, 1988). pSTAB (Fig. 6A) contains the *S. flexneri *pINV replicon (nucleotides 202,317–204,916 of pWR100; Buchrieser *et al.*, 2000) with *sacB‐neo^R^* to allow positive selection for the presence or absence of the plasmid. When subjected to plasmid loss assays in *S. flexneri*, the rate of pSTAB loss was similar to the rate of instability of pINV lacking the three TA systems (Fig. 6B and C). Furthermore, we tested colonies at random from our loss assays with pSTAB and examined them by PCR for the presence of the replicon as a marker for plasmid loss; results demonstrate that selection on sucrose accurately measures the number of plasmid‐free cells (data not shown).

To investigate the contribution each TA system has in plasmid maintenance, we inserted *ccdAB*, *gmvAT* and *vapBC *individually into pSTAB and monitored plasmid retention in *S. flexneri *lacking pINV (Fig. 6B and C). In comparison to pSTAB without any inserted sequence, we observed that *ccdAB* slightly increased maintenance of pSTAB in *S. flexneri* at 37°C but not 21°C (Fig. 6B and C, *p* = 0.0409 and *p* = 0.1822 respectively). As expected, VapBC had a dramatic effect on pSTAB, increasing stability by approximately two or three orders of magnitude at 37 and 21°C respectively (Fig. 6B and C, *p* < 0.0001). Insertion of *gmvAT* into pSTAB increased plasmid maintenance at 21°C by over two orders of magnitude in comparison to the empty vector (*p* < 0.0001). However, at 37°C, the effect of GmvAT on plasmid stability was not significant (*p* = 0.0888). We also conducted these experiments in a strain lacking the Lon protease, which would be expected to reduce the effectiveness of the TA systems. As predicted, the *lon* mutation completely abrogated the stabilising effect of the TA systems at both temperatures (Fig. 6B and C, *p* > 0.31) except for GmvAT, which still retained some, albeit greatly reduced, functionality in the Δ*lon* strain at 21°C alone (Fig. 6C, *p* = 0.0043). Taken together, these results confirm that CcdAB, GmvAT and VapBC found on pINV in *S. flexneri* are functional TA systems, and that GmvAT is most effective at temperatures found outside the human host, in agreement with our previous work (McVicker and Tang, 2016). Furthermore, the TA systems enhance the maintenance of pSTAB under the conditions tested, respond as expected to the absence of Lon and hence validate the use of pSTAB as a tool to measure plasmid stability.

### ParAB and StbAB are functional and influenced by temperature

**Figure 7:**
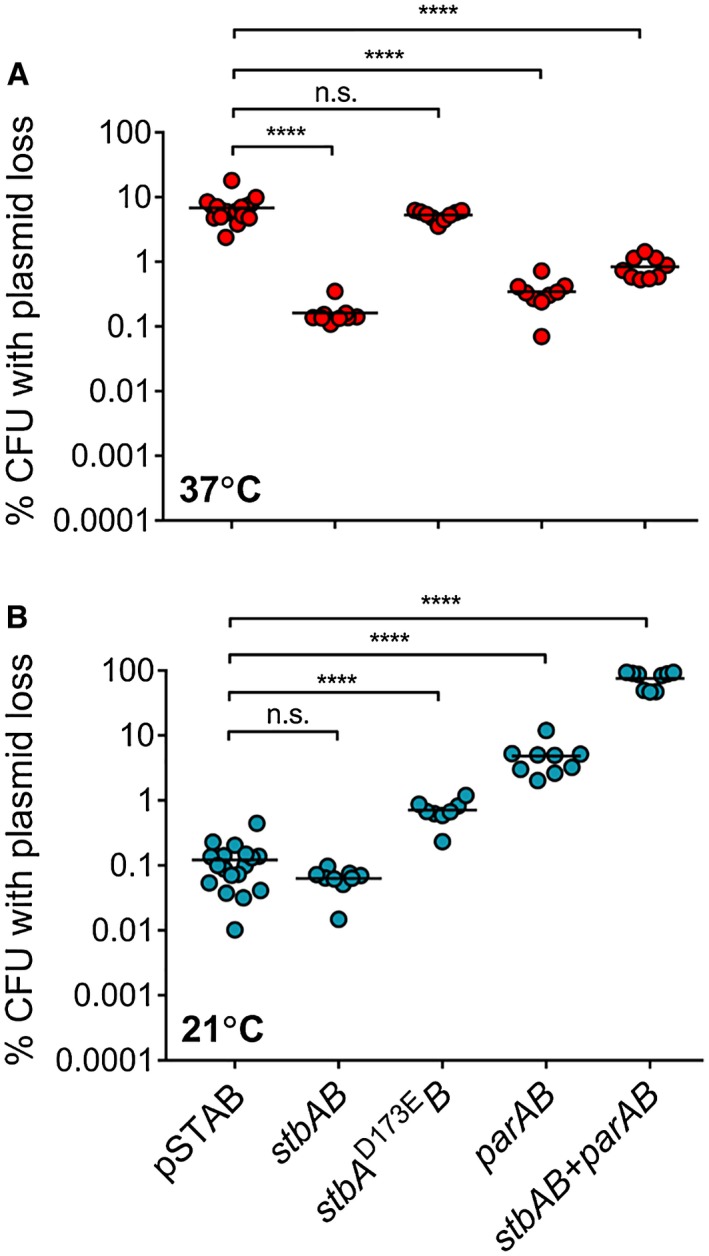
The effect of partitioning systems on pSTAB stability in *S. flexneri‐*lacking pINV. pSTAB loss measured by the *sacB‐neo^R^* assay with the plasmid carrying either no insertion, *stbAB*, *stbAB* encoding inactive StbA^D173E^, *parAB *or *stbAB*+*parAB*, grown at 37°C (A) or 21°C (B) for approximately 25 generations. ^****^ *p* < 0.0001; n.s. not significant by one‐way ANOVA with Dunnett multiple comparisons test (*n* ≥ 8 colonies from at least three independent experiments). [Colour figure can be viewed at wileyonlinelibrary.com].

We next examined whether the partitioning systems *parAB* and *stbAB *promote the stability of pSTAB by introducing these constructs into *S. flexneri *lacking pINV (Fig. 7). StbA contains an aspartic acid residue at position 173 that is conserved in ParM at position 170 (Supplementary Fig. 1) and is essential for ATPase activity; ParM is rendered non‐functional by D^170^E substitution (Jensen and Gerdes, 1997), therefore an inactive form of *stbAB *containing the equivalent D^173^E mutation was included as a control. At 37°C, results demonstrated that both *parAB* and *stbAB *stabilise pSTAB by approximately one order of magnitude over 25 generations of growth (Fig. 7A, *p* < 0.0001), whereas the inactive form of *stbAB* did not contribute to plasmid stability (Fig. 7A; *p* = 0.66 when compared with empty pSTAB). A version of pSTAB containing *stbAB* and *parAB* in tandem was also significantly stable relative to the empty vector (Fig. 7A, *p* < 0.0001).

We also examined the influence of *parAB* and *stbAB *on the stability of pSTAB in *S. flexneri *pINV^‐^ at 21°C (Fig. 7B). Consistent with *S. flexneri* pINV lacking the TA systems (McVicker and Tang, 2016), the stability of unmodified pSTAB was dependent upon temperature, with a significantly lower level of plasmid loss after 25 generations at 21°C than at 37°C (Fig. 7, *p* < 0.0001). This is not unprecedented, as at least one other T3SS‐encoding virulence plasmid replicon is controlled by temperature (Wang *et al.*, 2016). We also observed differences in the effect of the partitioning systems at 21°C compared with 37°C (Fig. 7). In contrast to 37°C, the presence of StbAB in isolation did not seem to affect the plasmid at 21°C (Fig. 7B, *p* = 0.8871), while the inactive version of StbA actually reduced plasmid stability at this temperature (Fig. 7B, *p* < 0.0001). The presence of ParAB alone was detrimental to plasmid stability at 21°C, causing an increase in plasmid loss of approximately two orders of magnitude (Fig. 7B, *p* < 0.0001), and insertion of *stbAB* and *parAB *in tandem resulted in even more dramatic instability (75.7% mean plasmid loss, *p* < 0.0001). Interestingly, we observed that there is a significant correlation between the size of sequence inserted into pSTAB and plasmid loss (Supplementary Fig. [Link], [Link], *r* ^2^ = 0.9787, *p* = 0.0107). The only operon insertion that does not fit this pattern is the wild‐type version of *stbAB*, indicating that this system contributes to plasmid segregation at this temperature. The nonfunctional *stbAB* D^173^E allele serves as an effective size‐matched control for this comparison.

Taken together, these results indicate that *S. flexneri* StbAB and ParAB are both active partitioning systems on pINV and that ambient temperature affects the function of pINV’s partitioning systems, similar to its TA systems (McVicker and Tang, 2016) and T3SS (Tobe *et al.*, 1995).

## Discussion

Large, low‐copy plasmids encode important virulence determinants in many enteric pathogens, including the four species of *Shigella *(reviewed by Pilla and Tang, 2018)*.* Whilst some plasmids are capable of moving horizontally from strain to strain, large, low‐copy virulence plasmids must also encode systems to ensure their vertical transmission from parent to daughter cells upon division, otherwise important traits would be lost.

The *Shigella* invasion plasmid, pINV, carries several functional TA systems and putative partitioning systems. In *S. flexneri*, two type II TA systems, VapBC and GmvAT, are required to stabilise the plasmid across the distinct temperatures experienced by this important human pathogen, which has to survive in the external environment as well as at the higher temperatures found within the human intestine (McVicker and Tang, 2016). Type II TA systems require the action of a specific protease to degrade the antitoxin (Muthuramalingam *et al.*, 2016). Many TA systems in enteric bacteria are governed by activity of the Lon protease (van Melderen *et al.*, 1994; Christensen and Gerdes, 2004; Hansen *et al.*, 2012; Winther and Gerdes, 2012). Therefore, we examined the impact of Lon and another antitoxin‐targeting protease, ClpP, on plasmid stability in *S. flexneri*. We found that deletion of Lon, but not ClpP, destabilised the virulence plasmid and abrogated the effect of mutations in the TA system loci, demonstrating that the action of the main *S. flexneri* pINV TA systems, VapBC and GmvAT, relies upon Lon (Figs 1 and 2). The impact of Lon on an acetyltransferase‐based TA system such as GmvAT has not been shown previously.

Lon is a housekeeping protease that has many distinct biological roles in prokaryotes and eukaryotes (van Melderen and Aertsen, 2009). Its N‐terminal domain is responsible for substrate recognition (Li *et al.*, 2005) and carries out ATP‐dependent unfolding and sequestration of substrates, activities that are divorced from its proteolytic activity (van Melderen and Gottesman, 1999). Of relevance, Lon substrates can be protected against degradation by binding to their ligands, as is the case for TA antitoxins like CcdA (van Melderen *et al.*, 1996). Lon influences many virulence traits in bacteria, including quorum sensing (Bertani *et al.*, 2007), motility, biofilm production (Claret and Hughes, 2000; Marr *et al.*, 2007) and the activity of T3SSs (Jackson *et al.*, 2004). Therefore, it is interesting that Lon is also necessary for enhanced stability of the *Shigella* invasion plasmid, with this housekeeping protease involved in the activity as well as retention of the T3SS.

Construction of plasmids lacking multiple maintenance systems is challenging as they become increasingly unstable, making it more difficult to perform each sequential genetic manipulation. Interpretation of the function of individual systems on a large plasmid is also complex as there are often multiple systems which might have redundant roles, and contain insertion sequences which can mediate localised deletions without plasmid loss (Pilla *et al.*, 2017); furthermore, pINV can spontaneously integrate into the chromosome *via* insertion sequences, affecting expression of genes on the PAI (Pilla *et al.*, 2017). To circumvent these problems, we constructed a test vector, pSTAB, which contains the origin of replication from pINV, as well as the same counter‐selectable marker we used to assay pINV stability previously (McVicker and Tang, 2016); experiments with pSTAB allowed us to delineate the function of each individual maintenance system.

The impact of partitioning systems ParAB and StbAB on pINV stability has not previously been analysed. Deletion of either system did not affect the stability of pINV (Fig. 3), suggesting they are functionally redundant, except that StbAB seemed to have a subtle but significant stabilising effect at 21°C in the absence of ParAB. Deletion of the regulatory protein VirB, which shares homology with ParB (Watanabe *et al.*, 1990), did not reduce pINV stability (Fig. 4).

To further evaluate the function of the partitioning systems required the use of pSTAB. We found that *parAB* and *stbAB* are capable of stabilising pSTAB by approximately one order of magnitude over 25 generations at 37°C (Fig. 7A), confirming ParAB function within a model system (Sergueev *et al.*, 2005) and providing evidence that StbAB is active.

**Figure 8:**
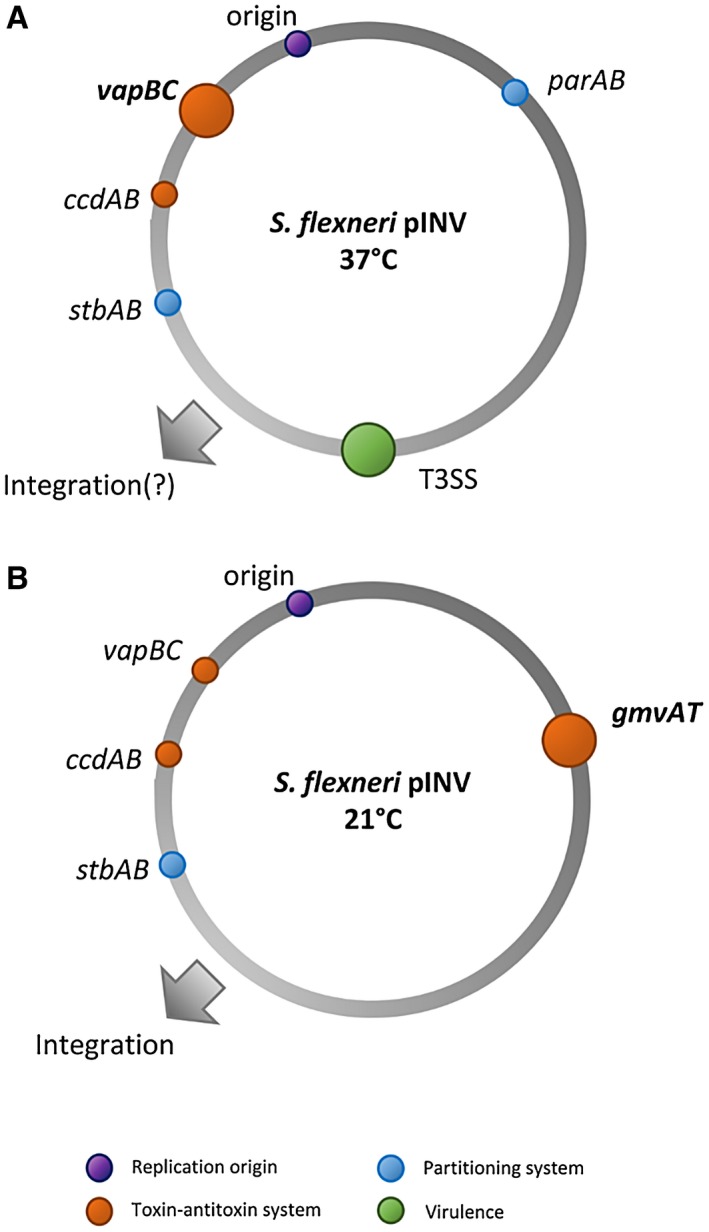
Temperature‐dependent functions of *S. flexneri *pINV maintenance systems. Position on the grey circle (pINV) shows the position of each operon. Appearance of a coloured dot indicates that the system is functional at the given temperature; larger dots with bold labelling indicate the main maintenance element at each temperature. Grey arrows reflect chromosomal integration data from Pilla *et al*. (2017). [Colour figure can be viewed at wileyonlinelibrary.com].

Similar to TA systems (McVicker and Tang, 2016), the stabilising effect of the partitioning systems is influenced by the ambient temperature. StbAB was most effective at 37°C and initially appeared dispensable at 21°C, while ParAB was functional at 37°C and actually destabilised the plasmid at 21°C (Fig. 7). The increased importance of these proteins at 37°C may reflect the higher growth rate, and thence the reliance on partitioning systems to ensure the rapid and faithful separation of plasmids into daughter cells. At lower temperatures, it is conceivable that cell division is slow enough to allow segregation, so partitioning systems are less important. The reason for the destabilising effect of ParAB at lower temperatures is not clear, although it is noteworthy that a non‐functional version of *stbAB* allele (based on Jensen and Gerdes, 1997) was also detrimental to pSTAB retention at 21°C, consistent with the effect on plasmid instability at 21°C being due to the change in size of the plasmid. Indeed, the plasmid with the largest insertion (i.e. *stbAB* and *parAB* in tandem) was dramatically unstable at 21°C, with instability directly correlated with insert size (Supplementary Fig. [Link], [Link], *r* ^2^ = 0.9787, *p* = 0.0107). This was not seen at 37°C, where instead, tandem insertion of the segregation systems stabilised pSTAB (Fig. 7A). Crucially, direct comparison of the effect of the functional and nonfunctional *stbA* alleles at 21°C indicates that StbAB is active at this temperature. This is consistent with the role of StbAB in pINV at 21°C requiring the absence of ParAB, as discussed above. Interestingly, an enteropathogenic *E. coli* virulence plasmid, pB171, also carries both type I and type II partitioning loci (Ebersbach and Gerdes, 2001), but these systems are adjacent and share a single regulatory region. In *Shigella* pINV, the two partitioning systems are encoded approximately 90 kb apart (Fig. 8) so there is unlikely to be direct crosstalk between ParAB and StbAB.

In this study, we provide evidence for the influence of global protease activity upon plasmid stability in *S. flexneri* and define further the maintenance systems encoded by pINV that operate at different temperatures (summarised with current knowledge in Fig. 8). We constructed a model vector using the pINV replicon that allows us to interrogate individual plasmid maintenance elements and have used it to confirm the temperature dependency of the TA systems, as shown previously (McVicker and Tang, 2016). Furthermore, we have demonstrated that while the partitioning systems StbAB and ParAB display redundancy, they are both functional at 37°C, with StbAB providing a degree of stabilisation at 21°C. This observation is important, since, like the TA system GmvAT that is solely functional at temperatures outside the human host, the *stbAB* operon is missing in *Shigella sonnei *(Supplementary Fig. 3). While the precise deletion has not been mapped, we could not detect *stbAB* in pINV of 132 sequenced *S. sonnei* isolates (Holt *et al.*, 2012), providing further evidence that this species is adapted to retain pINV at temperatures found inside the human body (McVicker and Tang, 2016).

## Experimental procedures

### Strains and growth media

The bacteria and plasmids used in this study are shown in Supplementary Table 1. *E. coli* and *Shigella *were propagated in liquid Lysogeny broth (LB; Invitrogen, Waltham, MA), or on solid media containing 1.5% (w/v) agar (Oxoid, Basingstoke, UK). Antibiotics were used at the following concentrations: carbenicillin, 50 µg ml^−1^; chloramphenicol, 20 µg ml^−1^; kanamycin, 50 µg ml^−1^. For *Shigella*, Congo red (0.01% w/v, final concentration) was added to tryptic soy broth (Fluka, Buchs, Switzerland) for solid media. For selection on sucrose, 1% (w/v) tryptone (Fluka), 0.5% (w/v) yeast extract (Fluka) and agar as above were autoclaved in water, with sucrose (final concentration of 10% w/v) added prior to pouring plates.

### Strain and plasmid construction

DNA manipulations were performed in *E. coli *DH5α and plasmids assembled using NEBuilder HiFi DNA Assembly master mix (New England Biolabs, Ipswich, MA). All primers (Supplementary Table 2) were purchased from Sigma. The *sacB‐neo^R^* cassette was amplified from pIB279 (Blomfield *et al.*, 1991). Electroporation was used to transform bacterial cells with plasmids or linear DNA. For insertion of mutations into the chromosome or pINV, λ Red recombination (Cherepanov and Wackernagel, 1995; Datsenko and Wanner, 2000) was performed using PCR products including ~1 kb of upstream and downstream flanking sequence. After λ Red recombination in *Shigella*, bacteriophage P1*vir* was used as previously described (McVicker and Tang, 2016) to transduce mutations into a clean genetic background to reduce the risk of off‐site mutations created by the λ Red system. All mutations were verified by PCR and sequencing.

### Virulence plasmid stability assays

Bacteria were grown on LB agar from frozen stocks, and incubated at different temperatures for ~25 generations (i.e. 25 doublings); after growth, whole colonies were re‐suspended in PBS, diluted, then plated to measure the number of bacteria harbouring pSTAB or pINV *mxiH*::*sacB‐neo^R^*, either on media containing kanamycin (pSTAB^+^ or PAI^+^, kanamycin resistant) or sucrose (pSTAB^−^ or PAI^−^) to detect the presence/absence of the *sacB‐neo^R^* cassette. The sum of these numbers was used to calculate the total number of CFUs to confirm the number of generations elapsed. Colonies were excluded from analysis if they had ~100% sucrose resistance (indicating a founder effect). Any difference in the growth rates of strains was accounted for by measuring the number of generations by assessing the number of CFU rather than using particular growth times.

### Statistical and computational methods

Data were log‐transformed (normally‐distributed) and analysed using unpaired *t*‐tests, linear regression, or by one‐way or two‐way ANOVA with appropriate multiple comparisons tests as indicated in figure legends. Statistical significance was assumed if *p* < 0.05.

## Author contributions

GM, SH and GP performed experiments and analysed data. GM, SH, GP and CMT designed experiments, interpreted data and wrote the manuscript. CMT secured funding.

## Conflict of interest

The authors declare no conflicts of interest.
