# Supplementary figures and images for: Maintenance of the virulence plasmid in Shigella flexneri is influenced by Lon and two functional partitioning systems
Source: Mol Microbiol. 2019 Mar 22;111(5):1355–66. doi: 10.1111/mmi.14225 (PMC6519299; doi:10.1111/mmi.14225)

## Slide 1
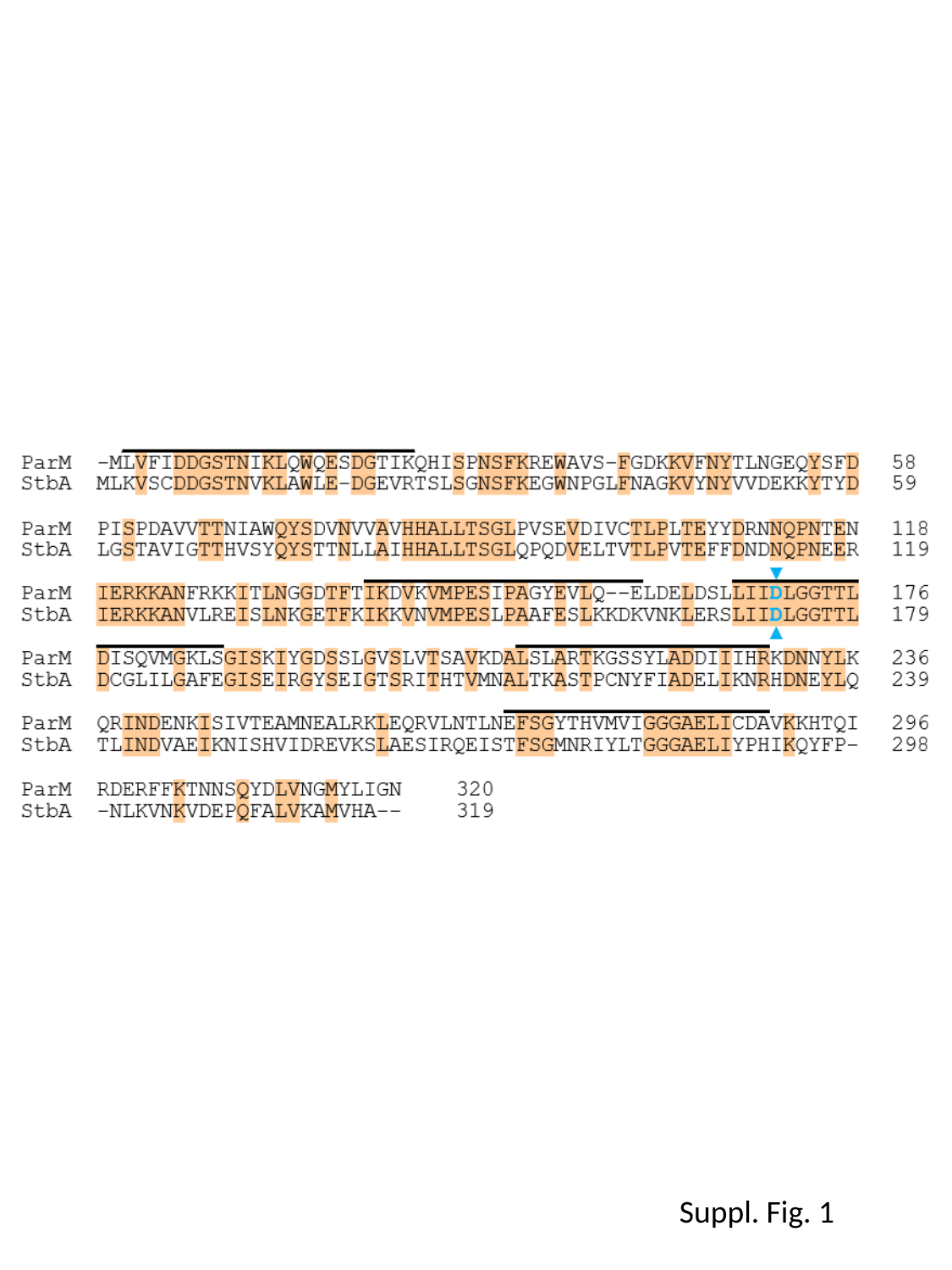

Suppl. Fig. 1

## Slide 2
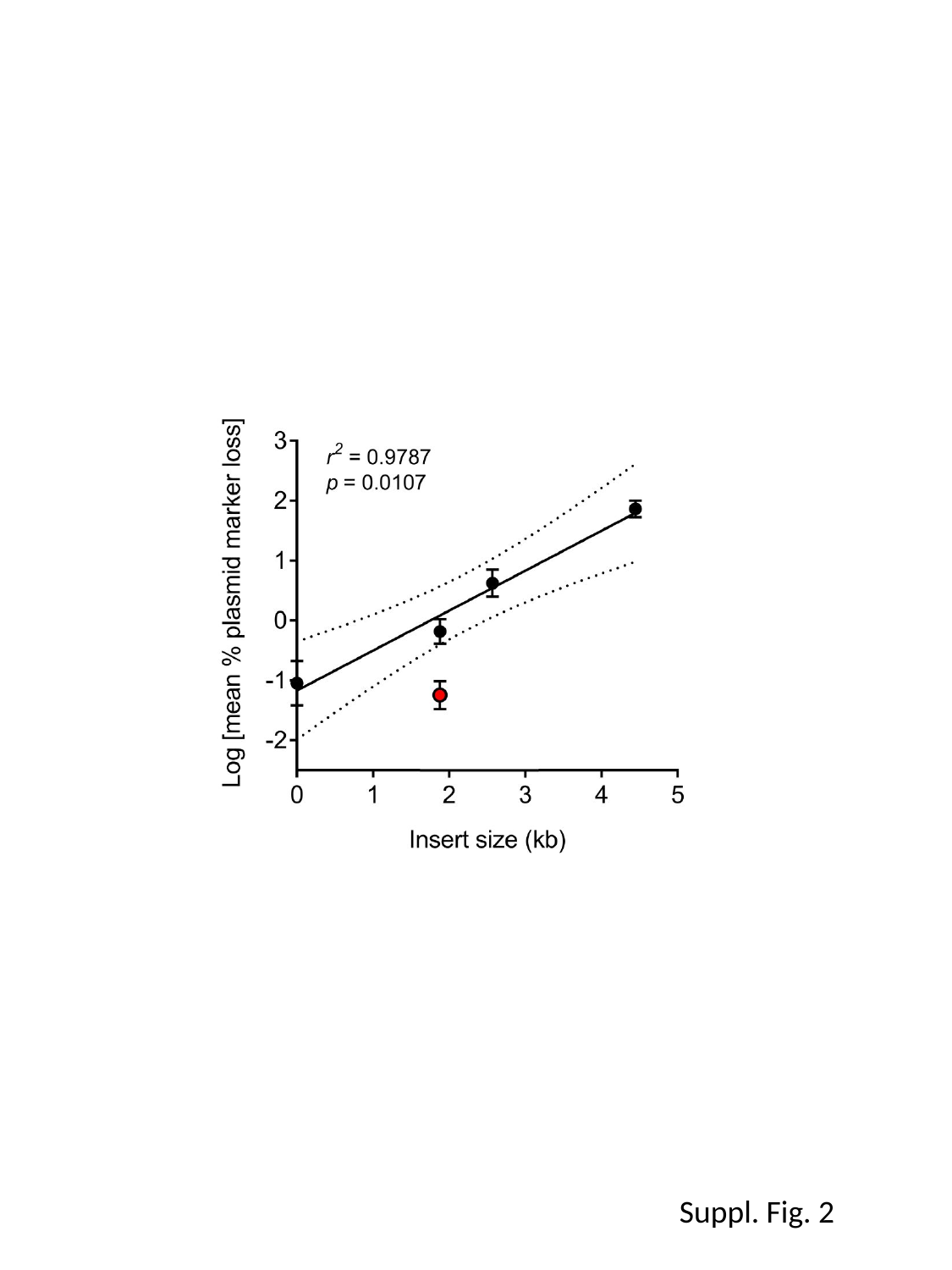

Suppl. Fig. 2

## Slide 3
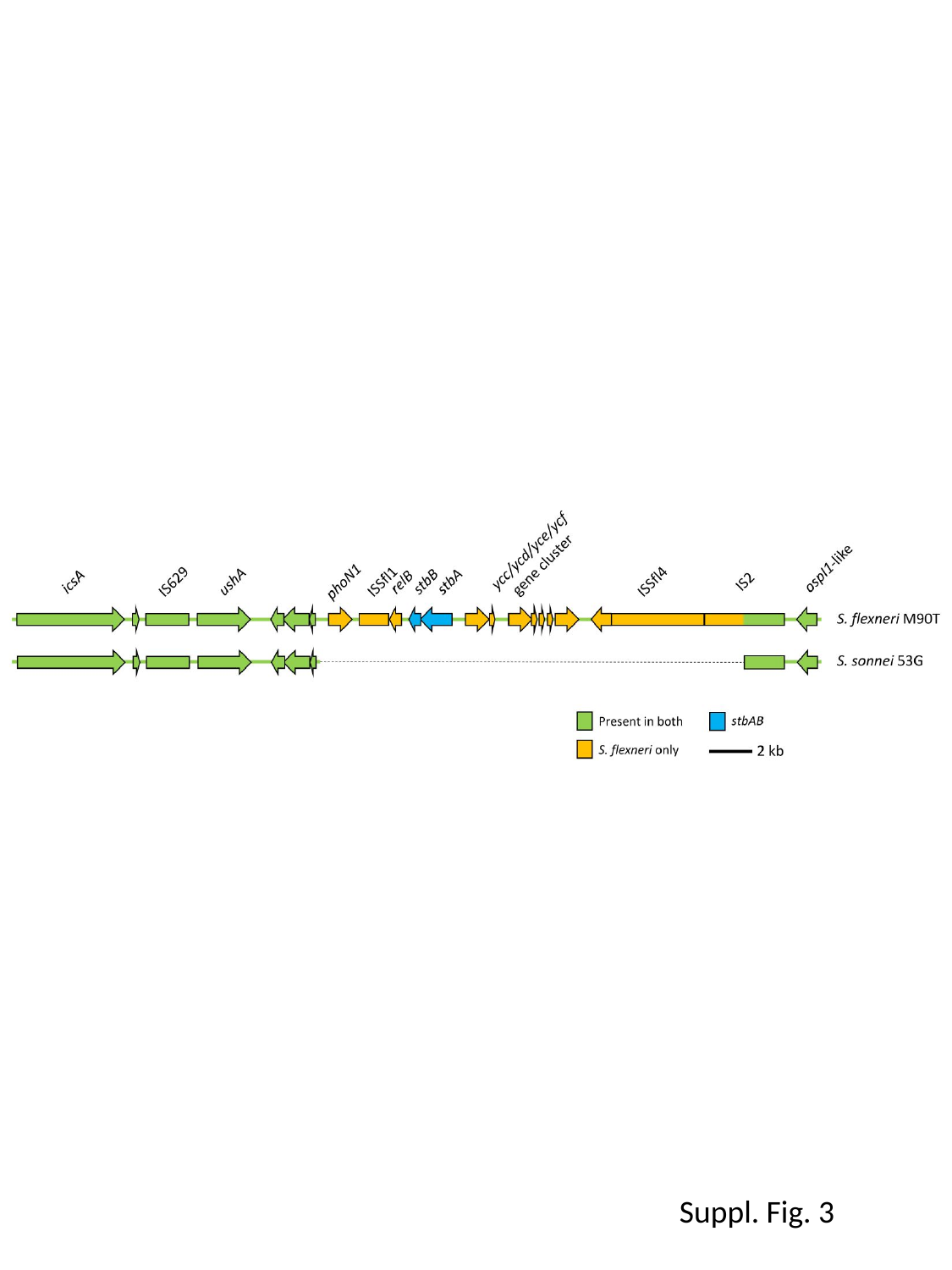

Suppl. Fig. 3

Supplement: Supplementary file 1 [file MMI-111-1355-s001.pptx]
