# Supplementary material for: Maintenance of the virulence plasmid in Shigella flexneri is influenced by Lon and two functional partitioning systems
Source: Mol Microbiol. 2019 Mar 22;111(5):1355–66. doi: 10.1111/mmi.14225 (PMC6519299; doi:10.1111/mmi.14225)
Supplement: Supplementary file 2 [file MMI-111-1355-s002.docx]

**Table 1: Bacterial strains and plasmids.**

| **Strain Name** | **Genotype** | **Reference** |
| --- | --- | --- |
| BS176 | pINV-cured *S. flexneri* M90T | Zychlinsky *et al.*, 1992 |
| GMCT189 | M90T *mxiH::sacB-neoR* | McVicker & Tang, 2016 |
| GMCT197 | M90T *mxiH::sacB-neoR* Δ*vapBC* | McVicker & Tang, 2016 |
| GMCT198 | M90T *mxiH::sacB-neoR* Δ*gmvAT* | McVicker & Tang, 2016 |
| GMCT208 | M90T *mxiH::sacB-neoR* Δ*ccdAB* Δg*mvAT* | McVicker & Tang, 2016 |
| GMCT209 | M90T *mxiH::sacB-neoR* Δ*ccdAB* Δ*vapBC* | McVicker & Tang, 2016 |
| GMCT213 | M90T *mxiH::sacB-neoR* Δ*ccdAB* Δ*gmvAT* Δ*vapBC* | McVicker & Tang, 2016 |
| GMCT253 | M90T *mxiH::sacB-neoR* Δ*clpP::cat* | This work |
| GMCT254 | M90T *mxiH::sacB-neoR* Δ*clpP::cat*  Δ*ccdAB* Δ*gmvAT* Δ*vapBC* | This work |
| GMCT255 | M90T *mxiH::sacB-neoR* Δ*lon::cat* | This work |
| GMCT256 | M90T *mxiH::sacB-neoR* Δ*lon::cat*  Δ*ccdAB* Δ*gmvAT* Δ*vapBC* | This work |
| GMCT266 | M90T *mxiH::sacB-neoR* Δ*vapBC* Δ*lon::cat* | This work |
| GMCT267 | M90T *mxiH::sacB-neoR* Δg*mvAT* Δ*lon::cat* | This work |
| GMCT268 | M90T *mxiH::sacB-neoR* Δ*ccdAB* Δg*mvAT* Δ*lon::cat* | This work |
| GMCT269 | M90T *mxiH::sacB-neoR* Δ*ccdAB* Δ*vapBC* Δ*lon::cat* | This work |
| GMCT356 | M90T *mxiH::sacB-neoR ΔstbAB::cat* | This work |
| GP_Sf23 | M90T *mxiH::sacB-neoR ΔparAB::cat* | This work |
| GMCT365 | M90T *mxiH::sacB-neoR ΔstbAB::cat*  Δ*ccdAB* Δ*gmvAT* Δ*vapBC* | This work |
| GP_Sf24 | M90T *mxiH::sacB-neoR ΔparAB::cat*  Δ*ccdAB* Δ*gmvAT* Δ*vapBC* | This work |
| GMCT418 | M90T *mxiH::sacB-neoR ΔparAB::cat* | This work |
| GMCT423 | M90T *mxiH::sacB-neoR ΔparAB::cat ΔstbAB* | This work |
| GP_Sf31 | M90T *mxiH::sacB-neoR ΔvirB::cat* | This work |
| GP_Sf32 | M90T *mxiH::sacB-neoR ΔstbAB ΔvirB:cat* | This work |
| GP_Sf35 | M90T *mxiH::sacB-neoR ΔparAB ΔvirB:cat* | This work |
| M90T | *S. flexneri* 5a wild type | Sansonetti *et al.*, 1982 |
| **Plasmid** | **Purpose/Genotype** | **Reference** |
| pCP20 | FLP recombinase for λ Red cassette excision | Cherepanov & Wackernagel, 1995 |
| pGM235 | pSTAB | This work |
| pGM236 | pSTAB2-*stbAB* | This work |
| pGM237 | pSTAB2-*parAB* | This work |
| pGM238 | pSTAB2-*vapBC* | This work |
| pGM239 | pSTAB2-*stbA*^D173E^*B* | This work |
| pGM241 | pSTAB2-*stbABparAB* | This work |
| pSH_ccd | pSTAB2-*ccdAB* | This work |
| pSH_gmv | pSTAB2-*gmvAT* | This work |
| pIB279 | Source of *sacB-neo^R^* cassette | Blomfield *et al.*, 1991 |
| pKD3 | Source of *camR* cassette for λ Red recombination | Datsenko & Wanner, 2000 |
| pKD46 | Helper plasmid for λ Red recombination | Datsenko & Wanner, 2000 |

**REFERENCES**

Blomfield, I.C., Vaughn, V., Rest, R.F., and Eisenstein, B.I. (1991). Allelic exchange in *Escherichia coli* using the *Bacillus subtilis sacB* gene and a temperature‐sensitive pSC101 replicon. *Mol Microbiol*, 5(6), 1447-57.

Cherepanov, P.P., and Wackernagel, W. (1995). Gene disruption in *Escherichia coli*: TcR and KmR cassettes with the option of Flp-catalyzed excision of the antibiotic-resistance determinant. *Gene*, 158(1), 9-14.

Datsenko, K.A., and Wanner, B.L. (2000). One-step inactivation of chromosomal genes in *Escherichia coli* K-12 using PCR products. *Proc Natl Acad Sci U S A*, 97(12), 6640-5.

McVicker, G., and Tang, C.M. (2016). Deletion of toxin–antitoxin systems in the evolution of *Shigella sonnei* as a host-adapted pathogen. *Nat Microbiol,* 2, 16204.

Sansonetti, P. J., Kopecko, D. J. and Formal, S. B. (1982). Involvement of a plasmid in the invasive ability of *Shigella flexneri*. *Infect Immun,* 35(3), 852-860.

Zychlinsky, A., Prevost, M. C. and Sansonetti, P. J. (1992). *Shigella flexneri* induces apoptosis in infected macrophages. *Nature,* 358(6382), 167-169.
