# Supplementary material for: Maintenance of the virulence plasmid in Shigella flexneri is influenced by Lon and two functional partitioning systems
Source: Mol Microbiol. 2019 Mar 22;111(5):1355–66. doi: 10.1111/mmi.14225 (PMC6519299; doi:10.1111/mmi.14225)
Supplement: Supplementary file 3 [file MMI-111-1355-s003.docx]

**Supplementary Table 2: Oligonucleotide primers.**

| **Primer** | **5'-3' Sequence** | **Construct** |
| --- | --- | --- |
| GM174 | TGTGTAGGCTGGAGCTGCTT | *cat* cassette |
| GM175 | ATGGGAATTAGCCATGGTCC | *cat* cassette |
| GM234 | ttcctccaagttcGATAATCAGGCTGCGTTC | pGM239 |
| GM235 | cctgattatcgaaCTTGGAGGAACCACTCTG | pGM239 |
| GM241 | acggccagtgaattcgagctcGCTCATACAACTGCCAGC | Δ*stbAB::cat* |
| GM242 | ccatggctaattcccatTATTTAACCAGGCCGTGC | Δ*stbAB::cat* |
| GM243 | agcctacacaGCAACAGACAGGAATGCAATAATTTTG | Δ*stbAB::cat* |
| GM244 | ctatgaccatgattacgccaagcttCGAACTGGCTTTCATGAGC | Δ*stbAB::cat* |
| GM261 | acggccagtgaattcgagctcGAAGGCGGTAAAGCGTCTG | Δ*clpP::cat* |
| GM262 | ccatggctaattcccatTTCCGTCTCCTGGATAAAATTG | Δ*clpP::cat* |
| GM263 | agcctacacaCATCGTAATTGATGCCAGAGG | Δ*clpP::cat* |
| GM264 | ctatgaccatgattacgccaagcttCGCTTGCTTTGTCGGACT | Δ*clpP::cat* |
| GM267 | acggccagtgaattcgagctCATCATTCAGAAGCTGTTG | Δ*lon::cat* |
| GM268 | ccatggctaattcccatAGAGCTCTCTCTTAGTTTAATTTC | Δ*lon::cat* |
| GM269 | agcctacacaTGACCTCGCGCAAAATGC | Δ*lon::cat* |
| GM270 | ctatgaccatgattacgccaagcttCCCAGTTTCAGCTCACGAG | Δ*lon::cat* |
| GM383 | gcgataggaattaaaaccgccatggccatatggctagcatgagcggatccgaattcgagctccgtcgacaagcttgcggccgcactcgagcaccGATCCTTTTTAACCCATCACATATAC | pGM235 |
| GM384 | tcagcaggaaaCTTGGCGCAAACGTTGATTG | pGM235 |
| GM385 | ttgcgccaagtTTCCTGCTGAACATCAAAGGCAAG | pGM235 |
| GM386 | caactgagcaacgcgaaggcTGCAAGCCTCGTCGTCCTG | pGM235 |
| GM387 | GCCTTCGCGTTGCTCAGTTGTCCAACCCCGG | pGM235 |
| GM388 | ggtgctcgagtgcggccgcaagcttgtcgacggagctcgaattcggatccgctcatgctagccatatggccatggcGGTTTTAATTCCTATCGCTCAAG | pGM235 |
| GM389 | ccatatggctagcatgagcggatccAACCGGGCGACCGCATAC | pGM236 |
| GM390 | ggtgctcgagtgcggccgcaagcttACAATAATGCATATTTGCATTACAAGCAACGAG | pGM236 |
| GM391 | ccatatggctagcatgagcggatcCATTCCGATGATTAATAAATCAG | pGM237 |
| GM392 | ggtgctcgagtgcggccgcaagcttGTATCCCGCTTACTTTTG | pGM237 |
| GM393 | ccatatggctagcatgagcggatccGTAATTATCCTTGTCGGTATAATAATTACC | pGM238 |
| GM394 | ggtgctcgagtgcggccgcaagctTGAACAGGTCAGCTCCAG | pGM238 |
| GM406 | aaacgacggccagtgaattcgagctCCGTTATAAAACGACGCTTC | Δ*parAB::cat* |
| GM407 | catatggaccatggctaattcccatGAATATTACTGTAAGATAGTATGCC | Δ*parAB::cat* |
| GM408 | cttcgaagcagctccagcctacacaCTTCTTTGCTTGAAAAAGATAGTTTCTC | Δ*parAB::cat* |
| GM409 | aacagctatgaccatgattacgccaagcttCAAGAGCCGTATGACGCG | Δ*parAB::cat* |
| GM416 | catcggaatgACAATAATGCATATTTGCATTACAAGCAACGAG | pGM241 |
| GM417 | gcattattgtCATTCCGATGATTAATAAATCAG | pGM241 |
| GP130 | CCAGTGAATTCGAGCTCAACTATGGAATTAGGTGCAAGAAGAAG | Δ*virB::cat* |
| GP131 | GGACCATGGCTAATTCCCATCATCGAACACTGATGTAAACTGCCC | Δ*virB::cat* |
| GP132 | GTTTACATCAGTGTTCGATGATGGGAATTAGCCATGGTCCATATG | Δ*virB::cat* |
| GP133 | CTATGGAGCTCTCACATCAGAGTGTGTAGGCTGGAGCTGCTT | Δ*virB::cat* |
| GP134 | GCAGCTCCAGCCTACACACTCTGATGTGAGAGCTCCATAGATG | Δ*virB::cat* |
| GP135 | GCTATGACCATGATTACGCCAAGCTCCATACTCCAGATAATTCGG | Δ*virB::cat* |
| SH20 | ccatatggctagcatgagcggatccGCTGTGCCTGCTTTCATG | pSTAB2-*ccdAB* |
| SH21 | ggtgctcgagtgcggccgcaagcTTATATTCCCCAGAACATCAGG | pSTAB2-*ccdAB* |
| SH22 | ccatatggctagcatgagcggatcCACAATGTTCAGGTCTCTG | pSTAB2-*gmvAT* |
| SH23 | ggtgctcgagtgcggccgcaagctTCAGACTTTATAAAACAAGGTATTAG | pSTAB2-*gmvAT* |
